# Production of laccase enzyme from *Curvularia lunata* MY3: purification and characterization

**DOI:** 10.1007/s12223-023-01088-2

**Published:** 2023-09-11

**Authors:** Ahmed A. Hamed, Ahmed M. Abd-Elaziz, Manal M. E. Ghanem, Mohamed E. ElAwady, Mohamed S. Abdel-Aziz

**Affiliations:** 1 Microbial Chemistry Department, National Research Center, 33-El Bohouthst, P.O.12622, Dokki, Giza, Egypt; 2 Molecular Biology Department, National Research Center, 33-El Bohouthst, P.O.12622, Dokki, Giza, Egypt; 3 Department of Microbial Biotechnology, National Research Center, 33-El Bohouthst, P.O.12622, Dokki, Giza, Egypt

**Keywords:** Characterization, *Curvularia lunata*, Laccase, Purification

## Abstract

Laccase-producing fungus (MY3) was successfully isolated from soil samples collected from Mansoura Governorate, Egypt. This fungal isolate has shown a high laccase production level over other isolated fungi. The identity of this isolate was determined by the molecular technique 18SrRNA as *Curvularia lunata* MY3. The enzyme purification was performed using ammonium sulfate precipitation followed by Sephacryl S-200 and DEAE-Sepharose column chromatography. The denatured enzyme using SDS-PAGE had a molar mass of 65 kDa. The purified laccase had an optimum temperature at 40 °C for enzyme activity with 57.3 kJ/mol activation energy for 2,2′-azino-bis-3-ethylbenzothiazoline-6-sulfonic acid (ABTS) oxidation. The enzyme had an optimum pH of 5.0, and it has shown a high stability at the acidic range (4.5 to 5.5). Mn^2+^ and Mg^2+^ ions enhanced the enzyme activity, while most of the enzyme activity was inhibited by Hg^2+^. Some compounds such as 2-mercaptoethanol, L-cysteine, and sodium azide at a concentration of 10 mmol/L had shown a high suppression effect on the enzyme activity. The enzyme strongly oxidized ABTS and syringaldazine and moderately oxidized DMP and guaiacol. The antimicrobial activity of the purified enzyme towards three pathogenic strains (*Escherichia coli* ATCC-25922,* Staphylococcus aureus* NRRLB-767, and* Candida albicans* ATCC-10231) was evaluated for the potential use as an antimicrobial therapeutic enzyme.

## Introduction

Laccase enzyme (EC 1.10.3.2) is a cuproenzyme that oxidize various types of phenols and aromatic compounds (Moshtaghioun et al. 2011). Laccases are excessively found in nature and usually called multi-copper oxidases because they contain copper atoms in the catalytic center which condense molecular oxygen to water and jointly execute one electron oxidation of a variety of aromatic substrates (Messerschmidt et al. 1992; Nyanhongo et al. 2002). These copper atoms coordinate the electron transfer steps in the redox reactions (Kunamneni et al. 2008a, b). Despite laccases’ low substrate specificity, they play a putative role in various processes in nature due to their ability to oxidize a wide range of compounds without liberation of any toxic intermediate peroxide (Claus 2004; Nyanhongo et al. 2002).

Laccase enzyme has been produced from animals, insects, higher plants, fungi, and bacteria (Cavallazzi et al. 2005). But the most important sources of these enzymes are basidiomycetes (Kunamneni et al. 2008a, b). This makes them suitable for many biotechnological applications (Cavallazzi et al. 2005; Annuar et al. 2010). Fungal laccases have proven to be more potential than plant or bacterial laccases due to their higher redox potential (up to + 800 mV) (Brijwani et al. 2010).

Fungal laccases are mainly glycoproteins with monomeric, dimeric, or tetrameric structures. Glycosylation of laccases produced from fungi has been reported to play a critical role in proteolytic degradation susceptibility, thermal stability, and copper retention (Unuofin et al. 2019). This fungal laccase nature allows them to be involved in several biotechnological and industrial applications, such as degradation of lignin, de-coloration, and detoxification to pulp bleaching, potentially toxic phenol removal, biosensors, detergents, and washing powders, and synthesis of complex medicinal components in addition to heteromolecular dimers of antibiotics through phenolic oxidation and production of anticancer enzyme-catalyzed preparations (Anyanwutaku et al. 1994; Thurston 1994; Kunamneni et al. 2008a, b).

This research work is undertaken with the aim of isolating, purifying, and characterizing the laccase enzyme from *Curvularia lunata* MY3 liquid cultures and evaluates its antimicrobial and antitumoral activity.

## Materials and methods

### Microorganisms

The Gram-positive bacterial strain *Staphylococcus aureus* NRRLB-767, a Gram-negative bacterial strain *Escherichia coli* ATCC-25922, and fungal strains (*Candida albicans* ATCC-10231) were obtained from Microbial Chemistry Dept., National Research Center, Egypt.

### Chemicals

Laccase substrates—2,2′-azino-bis(3-ethylbenzothiazoline-6-sulfonic acid) ABTS, 2,6-dimethoxyphenol (DMP), and syringaldazine (SGZ)—were supplied by Sigma-Aldrich; USA. Czapek Dox agar was supplied by Sigma-Aldrich. Other chemicals that used in the present study were of analytical grade. Buffers were prepared according to Gomori (1955).

### Fungal isolation

Soil samples were collected from agriculture fields at Dekernis, Mansoura Governorate, Egypt, during June 2017. Soil was taken at 10 cm depth. Soil samples were firstly sieved and then dried for 5 days at 25 °C. The samples were kept after drying at 10 °C followed by fungal strains isolation. Enumeration of the microbes present in the soil was done by using a serial dilution-agar plating method. Serial dilution of soil suspension was prepared up to 10^−6^ dilution. Then, 0.1 mL of suspension from dilutions 10^−3^ to 10^−6^ was spread on sterile Czapek Dox (CD) agar plates that previously supplemented with 50 mg/L filter sterilized nalidixic acid and 200 mg/L chloramphenicol for bacterial growth inhibition. Fungal growth was observed after incubation of the plates for 2 days. The fungi isolated on culture medium from soil were further purified by using of spore suspension and streak method. Routinely, the purified fungal cultures were transferred by streaking every 6 to 8 days onto fresh CD agar plates. Three to four transfers of the fungal isolate on agar plates were carried out before inoculating the liquid culture medium (Abdel-Aziz and Hezma 2013).

### Qualitative screening of laccase-producing fungi

Laccase production was achieved by plating each fungal strain onto PDA plates supplemented with 0.02% guaiacol and 0.5% tannic acid that were sterilized separately and added to the medium just before solidification. Remazol Brilliant Blue R (RBBR), 0.01%, was added as an indicator and the plates were incubated at 30 °C for 7 days. The formation of reddish brown halo in plates supplemented with tannic acid and guaiacol, and dark purple in plates containing RBBR indicated a positive production and secretion of laccase enzyme (Senthivelan et al. 2019).

### Molecular identification the fungal isolate (MY3)

The molecular identification of potent laccase-producing fungus has been accomplished by DNA isolation, amplification (PCR), and sequencing of the ITS region. The primers ITS2 (GCTGCGTTCTTCAT CGATGC) and ITS3 (GCATCGATGAAGAACGCAGC) were used for PCR, while ITS1 (TCCGTAGGTGAACCTGCGG) and ITS4 (TCCTCCGCTTATTGATATGC) were used for sequencing. Montage PCR Cleanup Kit (Millipore) was used to purify PCR products from unincorporated PCR primers and dNTPs. Sequencing was performed by using Big Dye terminator cycle sequencing kit (Applied Biosystems, USA). Sequencing products were resolved on an Applied Biosystems model 3730XL automated DNA sequencing system (Applied Biosystems, USA). *Candida* sp. was used as a control.

### Microorganism and culture conditions

*Curvularia lunata* MY3 was cultivated on three different culture media of the following compositions: M1: potato dextrose broth; M2 (g/L): malt, 2.0; glucose, 2.0; NaNO_3_, 2.0; NaH_2_PO_4_, 0.26; MgSO_4_·7H_2_O, 0.5; CuSO_4_, 0.01; CaCl_2,_ 0.006_;_ ZnSO_4,_ 0.005; MnSO_4_·2H_2_O, 0.0009; and M3: rice media. Conical flasks (250 mL-volume) containing 50 mL of culture medium were inoculated with 10% spore suspension (10^8^ CFU). To examine the optimum incubation time, the inoculated flasks were incubated on a rotary shaker (150 rpm) for 10 days. Also, the influence of different incubation temperatures that ranged from 25 to 60 °C on the *Curvularia lunata* MY3 laccase production was also investigated. pH that ranged from 4.0 to 8.0 was used to study their impact on the enzyme production. After incubation under the optimum conditions, the mycelia were removed from the culture medium by centrifugation under cooling at 5000 rpm for 30 min. The culture supernatant was considered as the enzyme source.

### Enzyme assay and protein determination

The laccase activity from the culture media of *Curvularia lunata* MY3 was determined by the oxidation of ABTS (2,2′-azino-bis-3-ethylbenzothiazoline-6-sulfonic acid) substrate method (Bourbonnais et al. 1998). The reaction mixture was containing 10 mmol/L ABTS as a substrate, 50 mmol/L sodium acetate buffer (pH 5.0), and a suitable amount of enzyme. The mixture was incubated for 20 min at 37 °C. The concentration of the cation radical responsible for the intense blue-green color can be correlated to the enzyme activity and is read at 420 nm. One unit of laccase activity was defined as the amount of enzyme required to oxidize 1 µmol of ABTS per min at 37 °C (Aslam et al. 2012). Protein concentration of the crude and purified protein samples was determined by the dye-binding method of Bradford (1976).

### Purification of *Curvularia lunata* MY3 Laccase

#### Ammonium sulfate precipitation

Total proteins of the enzyme-containing crude extract were fractionated by diammonium sulfate precipitation at the final concentration of 80% (w/v) with continuous stirring under cooling. The precipitated proteins were collected by cooling centrifugation at 15,000g for 20 min. The precipitate was re-suspended in 2 mL of 50 mmol/L sodium acetate buffer, pH 5.6.

#### Sephacryl S-200 chromatography

Ammonium sulfate–isolated proteins were further purified on Sephacryl S-200 column (95 × 1.6 cm i.d.) previously equilibrated with 50 mmol/L sodium acetate buffer; pH 5.6. The enzyme was applied to the column at 20 mL/h flow rate and collected in 3 mL fractions. The active laccase enzyme fractions were pooled and saved for further purification step.

#### DEAE-Sepharose chromatography

DEAE-Sepharose column (15 × 1.6 cm i.d.) previously equilibrated with 50 mmol/L sodium acetate buffer; pH 5.6 is used as an additional step of enzyme purification in which the pooled Sephacryl S-200 active laccase fractions were applied. A stepwise gradient NaCl ranging from 0.0 to 0.4 mol/L was used to elute the adsorbed proteins at a flow rate of 60 mL/h and 5 mL collected fractions.

### Characterization of *Curvularia lunata* MY3 laccase

#### Molecular weight determination

Subunit molar mass of the enzyme was estimated by SDS-PAGE according to Laemmli (1970) on 10% (w/v) acrylamide separating gel under denaturing conditions using a Bio-Rad (USA) Mini-protean II electrophoresis cell (BioRad, USA). Prestained protein ladder that ranged from 14 to 175 kDa was used for molar mass determination.

#### Effect of pH

The effect of pH on the activity of *C. lunata* MY3 purified laccase was examined over the range pH from 3.0 to 7.0 using 100 mmol/L of each: sodium citrate buffer (pH 3.0 to 4.0), sodium acetate buffer (pH 4.0 to 5.5), and sodium phosphate buffer (pH 5.5 to 7.0) in the presence of substrate. The effect of pH on the stability of the *C. lunata* MY3 laccase was performed by incubating the enzyme at different pH ranging from 3.0 to 9.0 for 24 h prior to add substrate. The residual activities were determined for each assay.

#### Effect of temperature

To examine the effect of temperature on the activity of *C. lunata* MY3 laccase, the complete enzyme reaction mixtures were incubated at different temperatures ranging from 10 to 80 °C. The same ranges of temperatures were used for investigating the thermal stability of the purified enzyme; the enzyme was pre-incubated at each temperature for 30 min followed by cooling, adding the substrate, and measuring the remaining activity as previously described.

#### Effect of metal cations

Several metal cations have been tested to measure their stimulatory and inhibitory effects on *C. lunata* MY3 purified laccase activity at 10 mmol/L of final concentration. The metal cations included K^+^, Mg^2+^, Ba^2+^, Na^+^, Ni^2+^, Ca^2+^, Mn^2+^, Co^2+^, Zn^2+^, Hg^2+^, and Cu^2+^. To examine the effect of each cation, the enzyme was pre-incubated with the different cations individually for 30 min in 50 mmol/L sodium acetate buffer, pH 5.0, at room temperature prior to substrate addition.

#### Inhibition studies

*C. lunata* MY3 laccase activity was measured upon incubation with several inhibitors and compounds. *Curvularia lunata* MY3 laccase was incubated with urea, iodoacetic acid (IAA), dithiothreitol (DTT), 1,10-phenanthroline (phen), ethylenediaminetetraacetic acid (EDTA), β-mercaptoethanol (ME), L-cysteine, and sodium azide individually at a concentration of 1.0 and 10 mmol/L for 30 min at room temperature; then, the remaining enzyme activities were measured.

#### Substrate specificity

The relative activities of *C. lunata* MY3 purified laccase towards different substrates ABTS, DMP, SGZ, 2-methoxyphenol (guaiacol), and benzene-1,2-diol (catechol) were examined. The substrates were incubated with the enzyme at a final concentration of 10 mmol/L, in 50 mmol/L sodium acetate buffer, pH 5.0.

#### Antimicrobial activity

The antimicrobial activity of the purified *C. lunata* MY3 laccase enzyme (10 µg) was investigated toward three pathogenic strains comprising gram-positive bacteria (*Staphylococcus aureus* NRRLB-767), gram-negative bacteria (*Escherichia coli* ATCC-25922), and yeast (*Candida albicans* ATCC-10231) using agar diffusion method with some modification of Hamed et al. (2018). Ampicillin and gentamicin were used as antibacterial control for gram-positive and gram-negative bacteria, respectively. Therefore, nystatin was used as antifungal one.

#### Cytotoxic activity of laccase on human tumor cell lines

To investigate the effect of laccase on cell viability, MTT (3,4,5-dimethylthiazol-2-yl)-2–5-diphenyltetrazolium bromide) assay was performed. The formation of a purple color indicates the reduction of MTT by the mitochondrial dehydrogenase of intact cells to form a purple formazan product (Mosmann 1983). Three different cell lines (colon cell line, Caco-2; human hepatocellular carcinoma cell line, HepG-2; and breast cancer cell lineMCF-7) were plated in a 96-well and incubated for 24 h at 37 °C before the treatments. After 24 h of incubation, different concentrations of purified laccase enzyme were added for different time intervals (48 and 72 h). After the treatment, the content was carefully removed by aspiration followed by the addition of 100 µL of 0.5 mg/mL MTT in cell culture medium to each well and incubated for 2 h. To dissolve the formazan crystals formed, 100 µL of 10% sodium dodecyl sulfate (SDS) was added and incubated overnight at 37 °C for 12 h for dissolving of the formed crystals. The amount of formazan formed was measured at 560 nm using a microplate reader.

## Results

### Fungal isolation and qualitative screening for laccase production

**Fig. 1:**
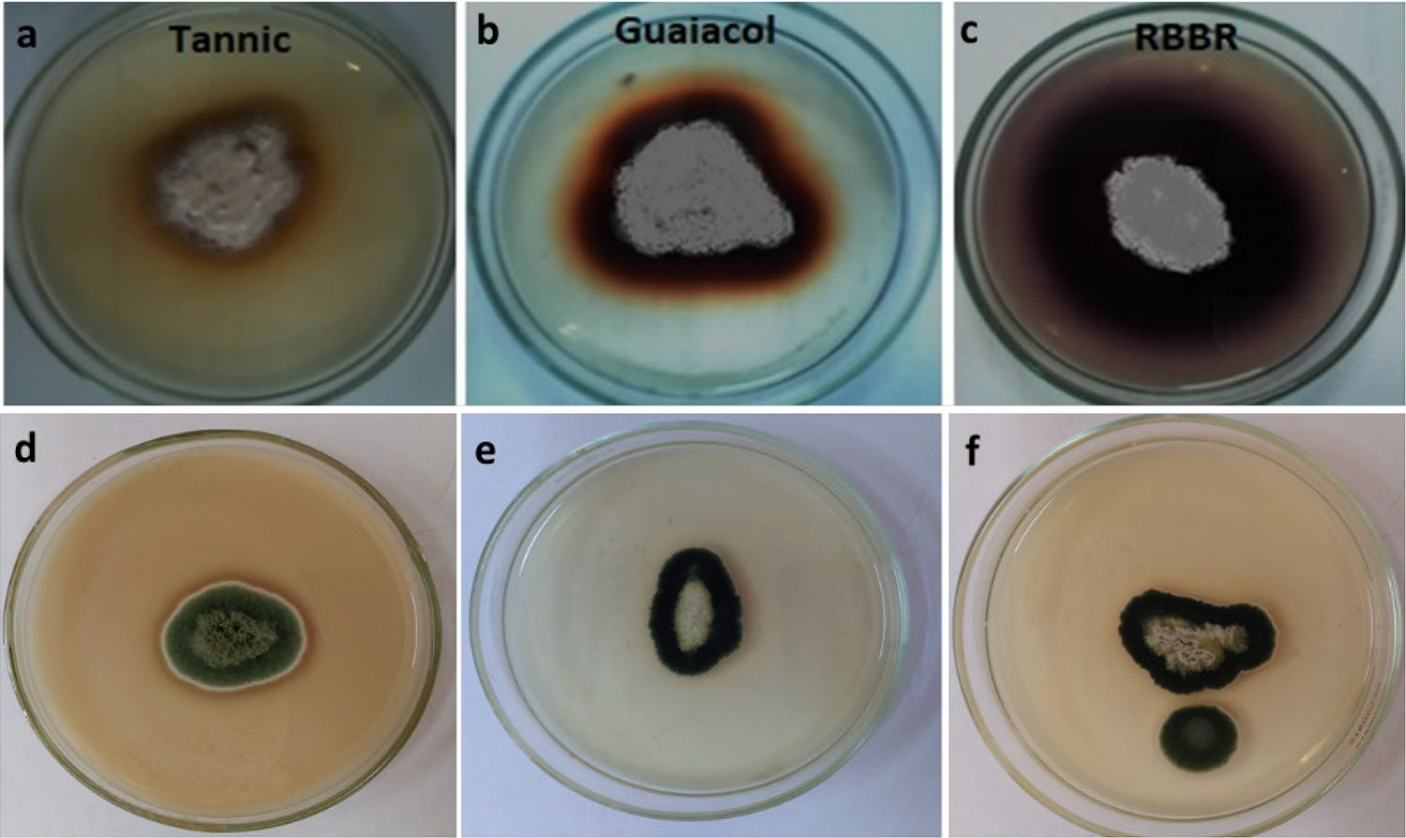
Qualitative screening of laccase producing fungi. Isolate MY3 grown on potato dextrose agar supplemented with tannic acid (**a**), guaiacol (**b**), and Remazol Brilliant Blue R (**c**), while **d**, **e**, and **f** represent the negative results

Five fungal strains were isolated (MY1 to MY5) from agriculture soils. The isolated strains were qualitatively screened by using agar plate medium, potato dextrose agar medium, supplemented with tannic acid, guaiacol and Remazol Brilliant Blue R, separately. After incubating the plates for the proper period (48–96 h), the appearance of colored halos around the fungal growth areas proved the laccase production. Results in Fig. 1 confirmed that the fungal strain MY3 has shown the maximum potent laccase production by appearance of reddish brown halos in case of tannic acid and guaiacol and dark purple color with Remazol Brilliant Blue R. Thus, the isolate MY3 was selected for further investigations including molecular identification, laccase production, laccase enzyme purification, and biochemical characterization.

#### Molecular identification of *Curvularia lunata* isolate MY3

**Fig. 2:**
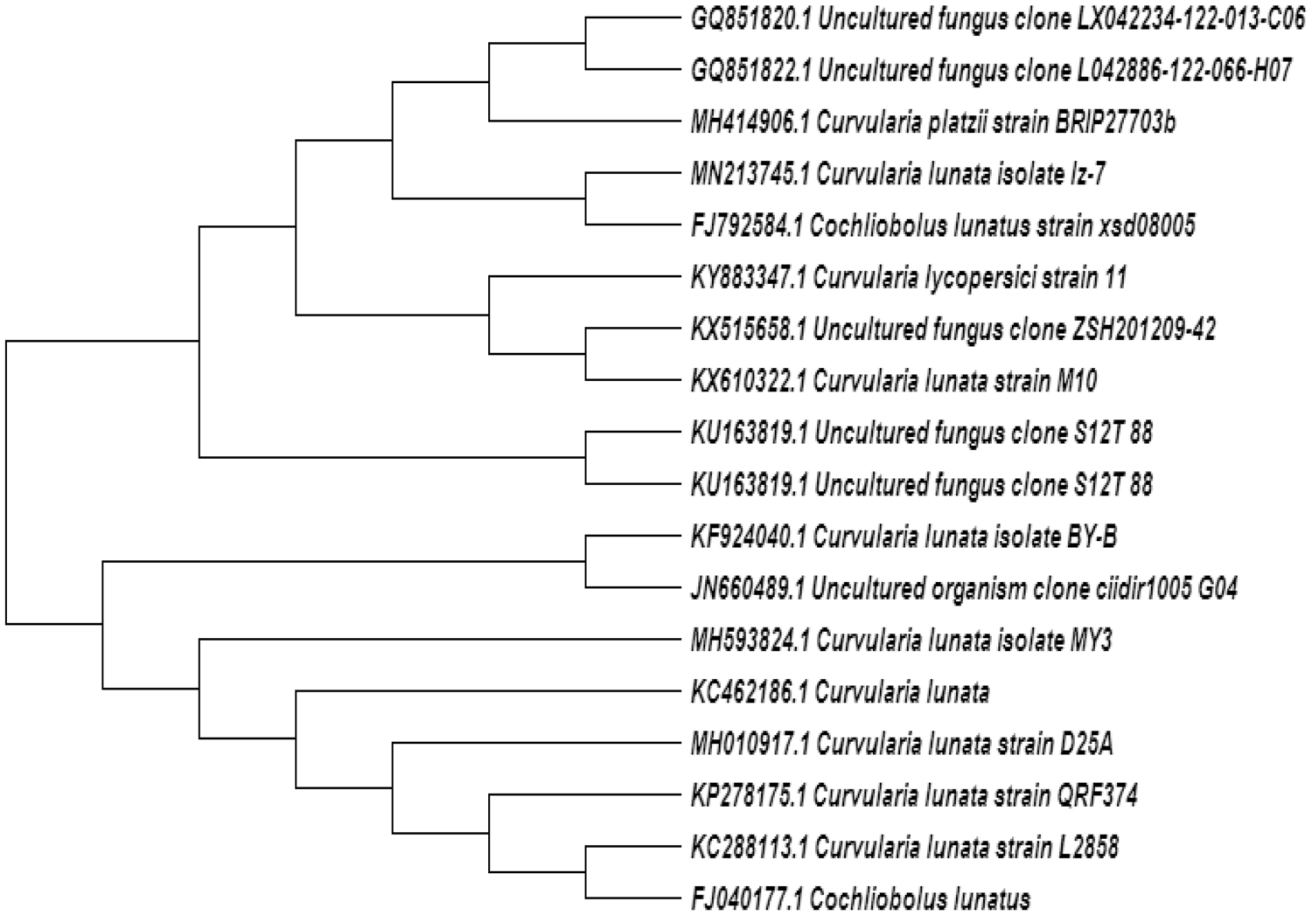
Phylogenetic trees showing relationship of strain *Curvularia lunata* isolate MY3 with other related fungal species retrieved from GenBank based on their sequence homologies of 18S rRNA

Nucleotide sequence of 594 bp of the whole 18SrRNA gene of the fungal sp. isolate MY3 was successfully determined. BLAST search of the identified sequence has revealed 100% similarity to *C. lunata* strain D25A (Acc. no. MH010917). The phylogenetic tree of this fungus was constructed (Fig. 2). The fungus was identified as *C. lunata* isolate MY3 with accession number (MH593824) and PubMed link: https://www.ncbi.nlm.nih.gov/nuccore/MH593824.

#### Production of laccase enzyme

**Fig. 3:**
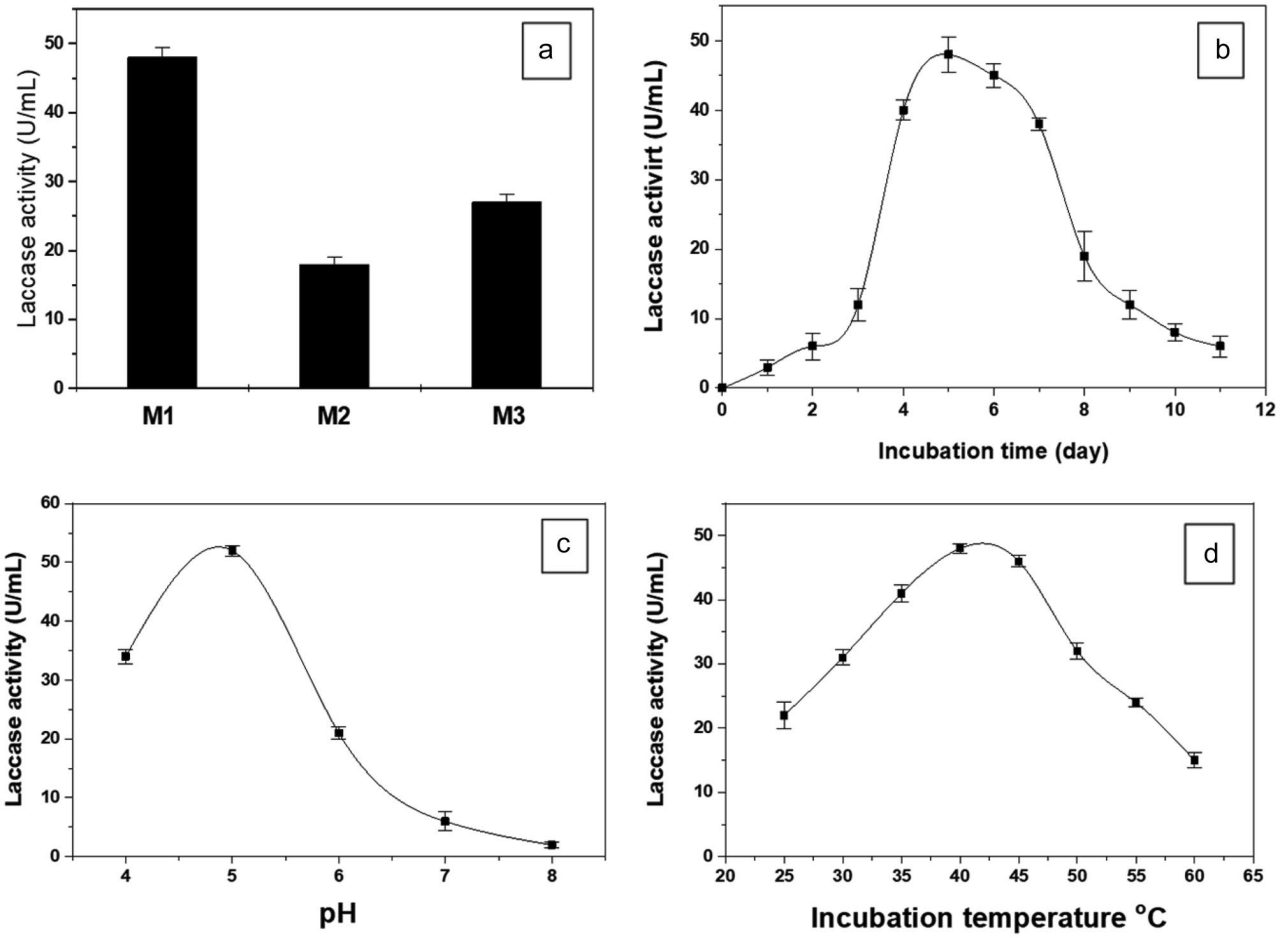
Physical parameters of amylase production: effect of different media (**a**), incubation period (**b**), different pH (**c**), and incubation temperature (**d**) on laccase production by fungal isolate MY3

The most potent laccase-producing isolate *C. lunata* MY3 has shown a high laccase activity by production of a brown halo around the fungal growth area on potato dextrose agar plates that previously supplemented with tannic acid (0.05% w/v). For further quantitatively screening, *C. lunata* MY3 was grown on three different media M1, M2, and M3. It has exhibited the highest laccase activity with M1 potato dextrose broth medium (Fig. 3a). The maximum laccase production was observed at the 5th day of incubation (Fig. 3b). The enzyme production was extremely reduced after the 6th day of incubation possibly may be as a result of cell death, the accumulative effect of the byproducts formed within the medium, and/or the exhaustion of nutrients.

The optimum pH for *C. lunata* MY3 laccase production was obtained at pH 5.0 (Fig. 3c). The maximum laccase production was achieved at 40 °C (Fig. 3d). The enzyme production was declined sharply above 45 °C due to the enzyme denaturation that resulted from the destruction of the secondary, tertiary, and quaternary bonds exist.

### Purification of *Curvularia lunata* MY3 laccase

**Fig. 4:**
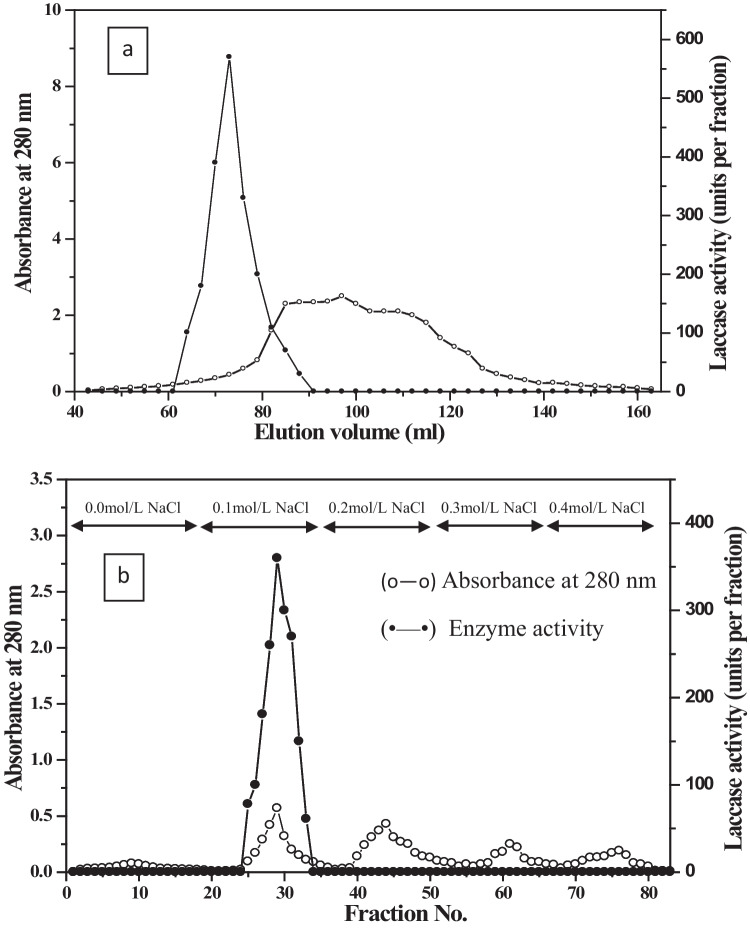
Elution profile for the chromatography of *C. lunata* MY3 laccase diammonium sulfate fraction on **a** Sephacryl S-200 column. **b** Sephacryl S-200 active fractions for *C. lunata* MY3 laccase on DEAE-Sepharose column. Absorbance at 280 nm (o^___^o) and laccase activity (●^____^●)

**Fig. 5:**
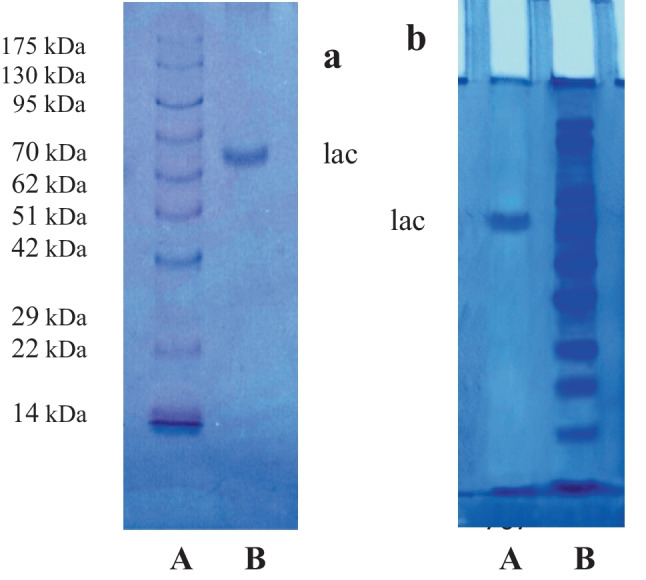
Electrophoretic analysis of *C. lunata* MY3 laccase. **a** Molar mass on 10% SDS-PAGE in (**A**) molar mass marker proteins and (**B**) purified laccase. **b** Native-PAGE in (**A**) purified laccase and (**B**) crude extract

The purification of laccase produced by *C. lunata* MY3 was performed using protein precipitation by ammonium sulfate followed by gel filtration and ion exchange chromatography. The crude filtrate was subjected to ammonium sulfate precipitation; then, the re-dissolved precipitate was dialyzed. After dialysis, further purification process was carried out using Sephacryl S-200 column (Fig. 4a). Fractions with the highest laccase activity were collected and then applied on a DEAE-Sepharose column for further purification (Fig. 4b). The majority of laccase enzyme was eluted with 0.1 mol/L NaCl with specific activity 1173.3 U/mg proteins with 8.5-fold purification. *C. lunata* MY3 laccase demonstrates a one single protein band on PAGE (Fig. 5b). The DEAE-active fractions were further subjected to gel filtration chromatography using Sephadex G-100 column.

### Characterization of *C. lunata* MY3 laccase

#### Molecular mass determination

SDS-PAGE analysis indicated that the protein isolated from *C. lunata* MY3 is a monomer where the enzyme migrated as a single protein band and was found to have a molar mass of 65 kDa (Fig. 5a).

#### Effect of temperature on enzyme activity and stability

**Fig. 6:**
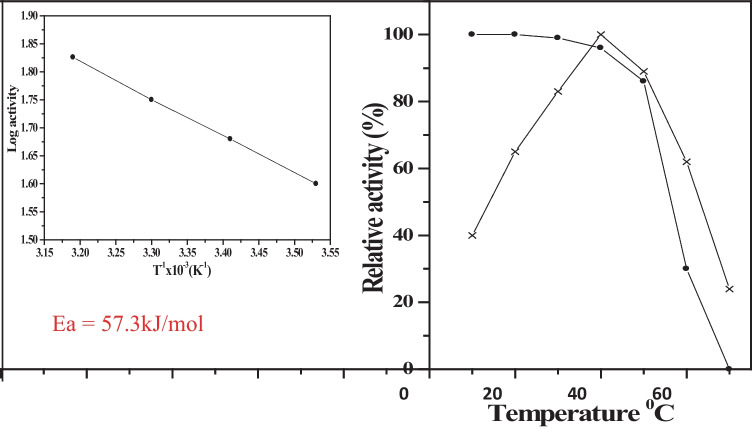
Temperature effects on the activity (x——x) and stability (•——•) of *C. lunata* MY3 laccase. Activation energy was estimated from Arrhenius plot shown in the inset

The enzymatic activity of *C. lunata* MY3 laccase was increased by increasing the temperature, and the optimal temperature for activity was observed at 40 °C. From 50 °C onward, the enzymatic activity declined where 75% of the activity was lost at 70 °C (Fig. 6). It was observed that *C. lunata* MY3 purified laccase maintain its activity in the range from 30 to 50 °C. At 60 °C onward, a dramatic decrease in the enzymatic activity is observed as the incubation progresses (Fig. 6).

#### pH optimum and stability

**Fig. 7:**
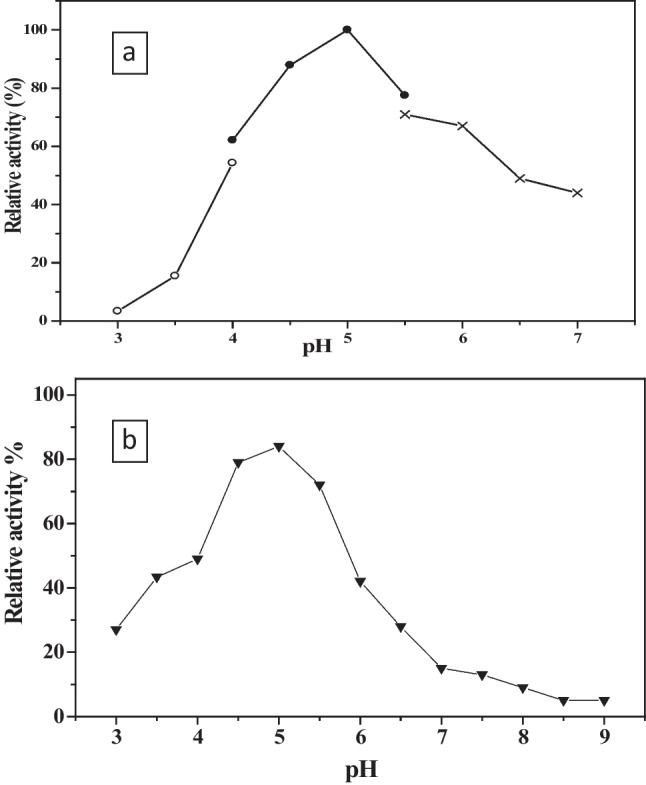
Effect of different pH on purified *C. lunata* MY3 laccase **a** activity and **b** stability: citrate buffer (o–o), acetate buffer (•-•), and phosphate buffer (x-x)

The results showed that *C. lunata* MY3 purified laccase can be classified as an acidic laccase since the optimal activity was achieved at pH 5.0. Moreover, the enzyme activity decreased beyond pH 5.0 (Fig. 7a). The stability profile of *C. lunata* MY3 purified laccase was detected at pH range from 4.5 to 5.5 as it retained about 83% of its activity upon incubation for 24 h. Its stability was inhibited from pH 6.0 onward (Fig. 7b). The characters of the purified laccase were compared with other purified laccases from different organisms (Table 1).

**Table 1:** Summary of the purified C. *lunata* MY3 laccase characters in comparison with other purified laccases from different organisms

**Organism**	**Molar mass in kDa**	**Optimum temperature in** °C	**Optimum pH**	**pH stability**	**References**
*Curvularia lunata* MY3	65	40	5.0	4.5–5.5	Present study
*Pleurotus ostreatus* HP-1	68	70	3.5		Patel et al. (2014)
*Pleurotus ostreatus* V184	65		3.0		Mansur et al. (2003)
*Pleurotus ostreatus* ARC280	85	50	3.0	8.0–10.0	Othman et al. (2014)
*Trametes orientalis*	50	80	4.0	4.5–5.5	Zheng et al. (2017)
*Trametes versicolor*	44	42.5	5.0	3.5–5.5	Brazkova et al. (2017)
*Trichoderma harzianum*	79	45	4.5		Sadhasivam et al. (2008)
*Marasmius* species BBKAV79	75	40	5.5		Vantamuri and Kaliwal (2016)
*Curvularia kusanoi*		40	6.0	3.75–5.75	Vázquez et al. (2019)

#### Effect of metal cations

**Fig. 8:**
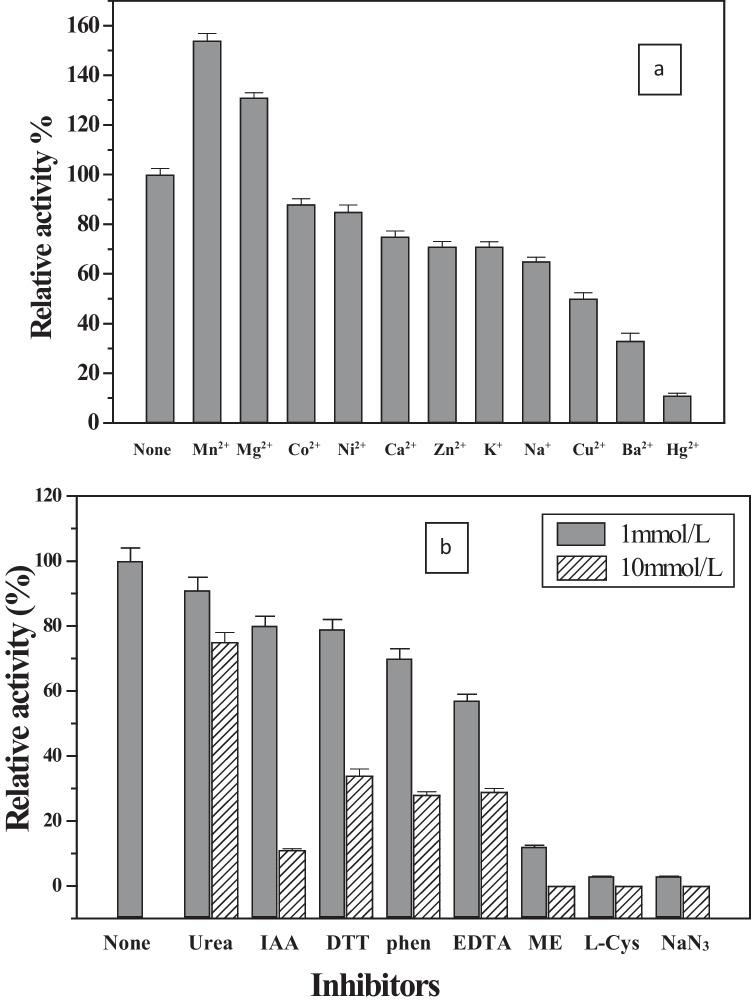
Effect of **a** 10 mmol/L of each metal cations and **b** different inhibitors on the activity of *C. lunata* MY3 laccase. The enzyme without any addition was taken as 100%

Upon incubation of *C. lunata* MY3 laccase with the examined metal cations, the enzyme activity was enhanced with manganese and magnesium ions by 154 and 131%, respectively. Except Mn^2+^ and Mg^2+^, all metal cations (10 mmol/L) had a suppression effect ranging from 22 to 89% inhibition of the enzyme activity (Fig. 8a). The effect of different concentrations of the most effective metals (Mn^2+^, Mg^2+^, Cu^2+^, Ba^2+^, and Hg^2+^) on the activity of laccase was estimated (Table 2). Complete inhibition was occurred at 200 mmol/L of each Ba^2+^ and Hg^2+^. The enzyme activity was activated with Mn^2+^ and Mg^2+^ at 200 mmol/L by 190 and 169%, respectively.

**Table 2:** Effect of different concentrations (10, 50, 100, and 200 mmol/L) of Mn^2+^, Mg^2+^, Cu^2+^, Ba^2+^, and Hg^2+^ on relative activity of purified laccase

**Metal**	**Concentration (mmol/L)**			
	**Relative activity (in %)**			
	**10**	**50**	**100**	**200**
Mn^2+^	154	172	183	190
Mg^2+^	131	148	160	169
Cu^2+^	45	32	14	2
Ba^2+^	37	15	4	Zero
Hg^2+^	11	3	Zero	Zero

The enzyme without any addition was taken as 100

#### Effect of inhibitors on *C. lunata* MY3 laccase activity

The sensitivity of *C. lunata* MY3 purified laccase towards different classes of inhibitors was investigated (Fig. 8b). It was incubated with different inhibitors for 30 min at final concentrations of 1 and 10 mmol/L. The enzyme was inhibited by all inhibitors in range from 9% to complete inhibition. No dramatic decrease in the enzyme activity had been observed at the concentration of 1 mmol/L EDTA, only 43% inhibition. By increasing EDTA concentration to 10 mmol/L, the activity was inhibited by about 71%.

#### Substrate specificity

Some of phenolic compounds were tested as possible substrates for *C. lunata* MY3 purified laccase. *C. lunata* MY3 purified laccase strongly oxidized ABTS (100%) and SGZ (91%), while moderately oxidized DMP (35%), guaiacol (52%), and catechol (38%).

#### Antimicrobial activity

The antimicrobial activity of the* C. lunata* MY3 purified laccase was assayed against a set of microorganisms using the agar diffusion technique. The antibacterial activity of purified laccase enzyme revealed that the purified enzyme displayed a potent antibacterial activity compared with gentamycin against Gram-positive *Staphylococcus aureus* NRRLB-767 with inhibition zone (17 mm) compared with ampicillin (21 mm), while there was no detected activity against Gram-negative *Escherichia coli* ATCC-25922 compared with gentamicin (20 mm). On the other hand, the purified laccase displayed a potent antifungal activity against *Candida albicans* ATCC-10231 when compared with nystatin with inhibition zone (18 mm).

#### Anticancer activity

In vitro cytotoxic effect of* C. lunata* MY3 purified laccase was examined toward three human tumors cell lines (Caco-2, HEPG2, and MCF7) in comparison with doxorubicin as reference control. The result showed that the purified laccase enzyme has antiproliferative activity that ranged from potent cytotoxic activity against HEPG2 with IC_50_ (13.68 µg/mL) to moderate cytotoxic activity against MCF7 and Caco-2 with IC_50_ (40.27 and 25.97 µg/mL) respectively (Table 3).

**Table 3:** In vitro cytotoxicity IC_50_ of C. *lunata* MY3 purified laccase against different cell lin

**Compound**	**In vitro cytotoxicity**		
	**HePG-2**	**MCF-7**	**Caco-2**
**DOX**	4.50 ± 0.2	4.17 ± 0.2	5.23 ± 0.3
**Laccase**	13.68 ± 0.6	40.27 ± 0.5	25.97 ± 0.6

*DOX* doxorubicin

## Discussion

One isolated strain MY3, in the maximum potent laccase production, from the five fungal strains (MY1 to MY5) was isolated from agriculture soils and was selected for further estimations including molecular identification, laccase production, laccase enzyme purification, and biochemical characterization. By using molecular identification, the fungus was identified as *Curvularia lunata* isolate MY3 with accession number (MH593824). A *Curvularia* sp. isolated from soil was found to contain laccase activity toward guaiacol as substrate. The organism produced an extracellular laccase in a medium containing yeast extract, peptone, and dextrose (Banerjee and Vohra 1991).

The maximum laccase production was estimated in a period from 5 to 7 days for *Botrytis cinerea* (Fortina et al. 1996) while other species of fungi required a longer time for laccase production (12–30 days). Elshafei et al. (2012) reported that fungal cultures prefer the acidic ranges of pH in the cultivation medium for biosynthesis of extracellular enzymes in agreement with the recent study. The production of laccase enzyme is, in fact, affected by the extracellular pH as it alters the cell permeability, inhibiting or changing the normal transport of the vital molecules, in addition to the conformational changes that occurred within the three-dimensional structure of the enzymes.

Different protocols have been employed for the purification of laccase enzyme. Laccase from *Trametes sanguinea* M85-2 was purified sixfold by chromatography on DEAE-Sepharose with 73% of final yield from the culture filtrate (Nishizawa et al. 1995). *Marasmius* sp*.* BBKAV79 cell-free supernatant was exposed to 80% ammonium sulfate salt precipitation and re-suspended in 50 mmol/L sodium acetate buffer (pH 5.5) then dialyzed against 50 mmol/L buffer for 12 h. The enzyme was then applied to Sephadex G-100 column followed by DEAE-cellulose column (Vantamuri and Kaliwal 2016). More et al. (2011) purified laccase enzyme from *Pleurotus* sp. by using ammonium sulfate precipitation followed by ion exchange chromatography on DEAE-cellulose column.

The molar mass of *C. lunata* MY3 laccase (65 kDa) was congruent to that recorded for previously purified laccase from solid-state fermented culture of *Pleurotus ostreatus* HP-1, 68 kDa (Patel et al. 2014), and comparable equal to that was isolated from *Abortiporus biennis* 66 kDa (Yin et al. 2017) and to the purified laccases LCC1 and LCC2 from* P. ostreatus* strain V-184 that possessed molecular weights of 60 and 65 kDa, respectively (Mansur et al. 2003). On the other hand, *C. lunata* MY3 laccase has a molar mass lower than that estimated for laccase from *P. ostreatus* ARC280, 85 kDa (Othman et al. 2014), and *Marasmius* species BBKAV79 and *Trichoderma harzianum*, 75 and 79 kDa, respectively (Vantamuri and Kaliwal 2016; Sadhasivam et al. 2008). *C. lunata* MY3 laccase has a molar mass higher than laccases purified from white rot fungus *Trametes orientalis* and *Trametes versicolor* 44 and 50 kDa, respectively (Zheng et al. 2017; Brazkova et al. 2017).

The optimal temperature for laccase activity of *C. lunata* MY3 (40 °C) is equal to the optimum temperature of recently purified laccase from *C. kusanoi* (Vázquez et al. 2019). Also, it is congruent to *Marasmius* species BBKAV79 40 °C, *T. Versicolor* 42.5 °C, and *Hericium coralloides* 40 °C (Vantamuri and Kaliwal 2016; Brazkova et al. 2017; Zou et al. 2012).* C. lunata* MY3 laccase optimum temperature for activity is lower by 1.25- to 2.0-folds than that recorded for *P. ostreatus* ARC280, *P. ostreatus* HP-1, and *T. orientalis* (Othman et al. 2014; Patel et al. 2014; Zheng et al. 2017). On the contrary, it is higher by 1.14-fold than that recorded for *T. harzianum* WL1 and *P. ostreatus* laccases (Sadhasivam et al. 2008; Palmieri et al. 1997). A wide range of these groups of oxidizing enzymes retain their activities over a wide range of temperatures from 5 to 55 °C. The behavior of thermal stability for *C. lunata* MY3 laccase was congruent with *C. kusanoi* L7 laccase (Vázquez et al. 2019), comparable to *Streptomyces cyaneus* laccase which is reported to have retained more than 75% of its activity after incubation for 120 min at 50 °C (Arias et al. 2003) and more than previously purified *P. ostreatus* EM-1 laccase that retains 22.6% of its initial activity upon incubation at 50 °C (Adamafio et al. 2012).

Laccases can have different optimum pH depending on the redox potential and the substrate used. In fact, fungal laccases are usually showed higher activity at acidic pH range from 3.5 to 5.0 when organic donors of hydrogen atoms are used as substrates, although pH stability varies considerably depending on the enzyme source. Such character had been explained through two effects: The first is the decrease in the solution ionization of the substrates with an increase in pH, and the second is the reduction of the hydroxyl ions binding with the active site of the enzyme that is associated with the increased pH values, resulting finally in decreasing the enzymatic-catalyzed reactions (Baldrian 2006; Zheng et al. 2017).

The acidic character of *C lunata* MY3 purified laccase (pH 5) was congruent with that reported for previously purified laccases from *Abortiporus biennis* J2 (Yin et al. 2017), *T. versicolor* (Brazkova et al. 2017), and *Thielavia* sp. (Mtibaà et al. 2018), where all showed the maximum enzyme activity at pH 5.0. Also, the pH profile of the present study is similar to that reported for other fungal laccases purified from *Trametes orientalis* pH 4.0 (Zheng et al. 2017), *T. harzianum* WL1, pH 4.5 (Sadhasivam et al. 2008), *Marasmius* sp. BBKAV79 pH 5.5 (Vantamuri and Kaliwal 2016), and* Curvularia kusanoi* L7 where the highest laccase activity was reached at pH 6.0 (Vázquez et al. 2019). The sharp decline in the enzyme stability above pH 6.5 might be a result of the exchange of the bonds that occurs in the alkaline and near alkaline conditions. These results are similar to laccases purified from *Trametes trogii* (Yan et al. 2014), *T. orientalis* (Zheng et al. 2017), and *Thielavia* sp. (Mtibaà et al. 2018). However, the high stability of *C. lunata* MY3 purified laccase in the acidic environment could be an interesting tool in biotechnological applications.

Similar effect of metal cations had been observed from other purified laccases by Othman et al. (2014) and Mtibaà et al. (2018). Desai and Nityanand (2011) reviewed the positive stimulatory effect of copper on microbial laccases and explained the possession of at least two types of laccase copper centers: one of mononuclear center serving as the oxidation site of the substrate, and the other is a trinuclear center where the reduction process of oxygen to water occurs. Despite this fact, not all laccases are stimulated by copper ions. For instance, incubation with copper ions had no stimulatory effect on laccases purified from *T. orientalis* and *Thielavia* sp. (Zheng et al. 2017; Mtibaà et al. 2018) in agreement with the laccase of the present study on *C. lunata* MY3. Hg^2+^ inhibited the activity of *C. lunata* MY3 laccase drastically at the concentration of 10 mmol/L. The same inhibition in laccase activity had been reported for *Xylaria* sp., *Marasmius* sp. BBKAV79, and *Sporothrix carnis* (Castaño et al. 2015; Vantamuri and Kaliwal 2016; Olajuyigbe and Fatokun 2017).

The chelating agent EDTA had severe inhibitory effect on most of purified fungal laccases (Ramírez-Cavazos et al. 2014). The moderate effect of EDTA on *C. lunata* MY3 purified laccase may be due to the low accessibility of EDTA to the structural atoms present in the active site of the enzyme which is responsible for the catalytic enzyme activity. The *C. lunata* MY3 laccase was drastically inhibited by DTT, L-cysteine, mercaptoethanol, and sodium azide. Similar behavior has been observed in other fungal laccases such as *Pleurotus* sp. (More et al. 2011), *Trametes orientalis* (Zheng et al. 2017), and *Aureobasidium pullulans* NAC8 (Ademakinwa and Agboola 2016). It was reported that NaN_3_ blocks the internal electron transfer and reduction of molecular oxygen by binding to the trinuclear copper center of the laccase enzyme, and the increase in its concentration resulted in abolishing the oxidation reaction catalyzed by the enzyme (Safary et al. 2016).

The enzyme affinity towards different substrates in addition to the rate of enzyme reaction was greatly depending on the substrate nature. So, there is a difference in terms of reactivity of the enzyme towards different compounds. The results are in congruent with that reported for *P. ostreatus* in which ABTS and SGZ were shown to be suitable substrates for laccases from all strains (Tinoco et al. 2001).

Laccases from *Fusarium oxysporum*, *Alternaria arborescence*, *Penicillium marneffei*, and *Aspergillus niger* exhibited antimicrobial activities against different G + ve and G-ve bacterial test microbes using disc diffusion method and kanamycin as standard (Christie and Shanmugam 2012). The proteinaceous nature of the laccase enzyme is the main reason of the antimicrobial activity. Study by Ibrahim et al. (2007) suggested that antimicrobial mechanism mediated by electrochemical mode of action that penetrates the cell wall of microbes causing disturbing of key cell function.

Several reports have mentioned the cytotoxic activity of laccases enzymes. The study conducted by Hu et al. (2011) showed that *Agrocybe cylindracea* purified laccase enzyme displayed a highly anti proliferative activity against HEPG2 cells and breast cancer. Another study conducted by Othman et al. (2014) showed that *P. ostreatus* ARC280 purified laccase exhibited cytotoxic activity against four human cell line HEPG2, MCF7, HTC116, and A549. From the obtained results and the previous reports, laccase enzymes could be useful in the therapy of different human tumors.

In conclusion, we screened several laccase producing fungal strains using PDA medium supplemented with guaiacol, tannic acid, and Remazol Brilliant Blue R. The strain *C. lunata* MY3 was identified and showed hyper-producing laccase ability. The produced enzyme was purified, characterized, and its antimicrobial activity was measured against three clinical test microbes. Interestingly, the enzyme showed potent antimicrobial activity toward *S. aureus* NRRLB-767 and* C. albicans*. Moreover, the purified enzyme exhibited antiproliferative activity against three cell lines, reflecting its possible use in the medical field.

## Data Availability

All data generated or analyzed during this study are included in this published article.

## Abbreviations

ABTS: 2,2′-Azino-bis(3-ethylbenzothiazoline-6-sulfonic acid
DMP: 2,6-Dimethoxyphenol
SGZ: Syringaldazine
CD: Czapek Dox agar
PDA: Potato dextrose agar
RBBR: Remazol Brilliant Blue R
ITS: Internal transcribed spacer
PCR: Polymerase chain reaction
IAA: Iodoacetic acid
DTT: Dithiothreitol
EDTA: Ethylenediaminetetraacetic acid
phen: 1,10-Phenanthroline
ME: β-Mercaptoethanol
SDS: Sodium dodecyl sulfate
